# Protocol for an open label: phase I trial within a cohort of foetal cell transplants in people with Huntington’s disease

**DOI:** 10.1093/braincomms/fcaa230

**Published:** 2021-01-19

**Authors:** Cheney J G Drew, Feras Sharouf, Elizabeth Randell, Lucy Brookes-Howell, Kim Smallman, Bernadette Sewell, Astrid Burrell, Nigel Kirby, Laura Mills, Sophie Precious, Philip Pallmann, David Gillespie, Kerry Hood, Monica Busse, William P Gray, Anne Rosser

**Affiliations:** Centre for Trials Research, Cardiff University, Cardiff, CF14 4YS, UK; Brain Repair and Intracranial Neurotherapeutics (BRAIN) Unit, Cardiff University, Cardiff, CF24 4HQ, UK; Brain Repair and Intracranial Neurotherapeutics (BRAIN) Unit, Cardiff University, Cardiff, CF24 4HQ, UK; Department of Neurosurgery, University Hospital Wales, Cardiff, CF14 4XW, UK; Centre for Trials Research, Cardiff University, Cardiff, CF14 4YS, UK; Centre for Trials Research, Cardiff University, Cardiff, CF14 4YS, UK; Centre for Trials Research, Cardiff University, Cardiff, CF14 4YS, UK; Swansea Centre for Health Economics, Swansea University, Swansea, SA2 8PP, UK; Public and Patient Representative, BRAIN Involve, Cardiff University, Cardiff, UK; Centre for Trials Research, Cardiff University, Cardiff, CF14 4YS, UK; Centre for Trials Research, Cardiff University, Cardiff, CF14 4YS, UK; Brain Repair Group, School of Biosciences, Cardiff University, Museum Ave, Cardiff, CF10 3AX, UK; Centre for Trials Research, Cardiff University, Cardiff, CF14 4YS, UK; Centre for Trials Research, Cardiff University, Cardiff, CF14 4YS, UK; Centre for Trials Research, Cardiff University, Cardiff, CF14 4YS, UK; Centre for Trials Research, Cardiff University, Cardiff, CF14 4YS, UK; Brain Repair and Intracranial Neurotherapeutics (BRAIN) Unit, Cardiff University, Cardiff, CF24 4HQ, UK; Brain Repair and Intracranial Neurotherapeutics (BRAIN) Unit, Cardiff University, Cardiff, CF24 4HQ, UK; Department of Neurosurgery, University Hospital Wales, Cardiff, CF14 4XW, UK; Neuroscience and Mental Health Research Institute, Cardiff University, Cardiff, CF24 4HQ, UK; Brain Repair and Intracranial Neurotherapeutics (BRAIN) Unit, Cardiff University, Cardiff, CF24 4HQ, UK; Brain Repair Group, School of Biosciences, Cardiff University, Museum Ave, Cardiff, CF10 3AX, UK; Neuroscience and Mental Health Research Institute, Cardiff University, Cardiff, CF24 4HQ, UK

**Keywords:** cell replacement therapy, Huntington’s disease, fetal cell, surgical intervention, trial within a cohort

## Abstract

Huntington’s disease is a progressive neurodegenerative disorder characterized by motor, cognitive and psychiatric symptoms. Currently, no disease-modifying therapies are available to slow or halt disease progression. Huntington’s disease is characterized by relatively focal and specific loss of striatal medium spiny neurons, which makes it suitable for cell-replacement therapy, a process involving the transplantation of donor cells to replace those lost due to disease. TRIal DEsigns for delivery of Novel Therapies in neurodegeneration is a phase I Trial Within a Cohort designed to assess safety and feasibility of transplanting human foetal striatal cells into the striatum of people with Huntington’s disease. A minimum of 18 participants will be enrolled in the study cohort, and up to five eligible participants will be randomly selected to undergo transplantation of 12–22 million foetal cells in a dose escalation paradigm. Independent reviewers will assess safety outcomes (lack of significant infection, bleeding or new neurological deficit) 4 weeks after surgery, and ongoing safety will be established before conducting each subsequent surgery. All participants will undergo detailed clinical and functional assessment at baseline (6 and 12 months). Surgery will be performed 1 month after baseline, and transplant participants will undergo regular clinical follow-up for at least 12 months. Evaluation of trial processes will also be undertaken. Transplant participants and their carers will be interviewed ∼1 month before and after surgery. Interviews will also be conducted with non-transplanted participants and healthcare staff delivering the intervention and involved in the clinical care of participants. Evaluation of clinical and functional efficacy outcomes and intervention costs will be carried out to explore plausible trial designs for subsequent randomized controlled trials aimed at evaluating efficacy and cost-effectiveness of cell-replacement therapy. TRIal DEsigns for delivery of Novel Therapies in neurodegeneration will enable the assessment of the safety, feasibility, acceptability and cost of foetal cell transplants in people with Huntington’s disease. The data collected will inform trial designs for complex intra-cranial interventions in a range of neurodegenerative conditions and facilitate the development of stable surgical pipelines for delivery of future stem cell trials. *Trial Registration:* ISRCTN52651778

## Teaser

The TRIDENT trial uses a ‘Trial Within a Cohort’ design to assess the safety and feasibility of escalating doses of foetal cells in participants with Huntington’s disease. This study focuses on addressing the many of constraints facing evaluation of advanced, non-pharmacological therapies in this and other neurodegenerative diseases.

## Introduction

Huntington’s disease is an inherited neurodegenerative condition characterized by progressive motor, cognitive and psychiatric deficits that significantly erode quality of life, resulting in a substantial societal cost (Jones *et al.*, 2016). Huntington’s disease is largely untreatable and no disease-modifying therapies currently exist. However, the relatively focal loss of medium spiny neurons in the striatum makes it a suitable candidate for cell-replacement therapy (CRT), in which donor cells are transplanted to replace those lost due to disease, with the expectation that the transplanted cells will re-establish some degree of normal neural circuitry.

An early target for CRT as a potential therapeutic strategy was Parkinson’s disease, with the aim of replacing degenerated mid-brain dopaminergic neurons. Transplants of human foetal-derived dopaminergic progenitors in Parkinson’s disease have been shown to produce significant and sustained benefit, establishing important proof of principle for CRT in general (Petit *et al.*, 2014; Barker *et al.*, 2015), albeit the key factors important for reliable benefit are still being explored through studies such as the ongoing Transeuro trial (Barker *et al.*, 2019). Early promise in Parkinson’s disease encouraged the exploration of CRT in Huntington’s disease. Here, the demands on the transplant are theoretically greater in that the transplanted tissue needs to reconstruct key elements of the host neural circuitry in order to provide functional benefit (Brasted *et al.*, 1999; Döbrössy and Dunnett, 2001; Mazzocchi-Jones *et al.*, 2009). This is in contrast to Parkinson’s disease where local release of dopamine may be sufficient to improve function. Thus, CRT in Huntington’s disease represents a more rigorous test of neural network reconstruction (Rosser and Svendsen, 2014).

Transplants of foetal striatal tissue in animal models of Huntington’s disease have demonstrated transplant survival, integration and alleviation of both functional motor and cognitive deficits (Dunnett *et al*., 2000). Subsequently, several open label trials transplanting human foetal striatal cells into limited numbers of Huntington’s disease participants have reported safety and feasibility [reviewed in (Rosser and Bachoud-Lévi, 2012)]. The most success to date comes from a pilot study of Bachoud-Lévi *et al*. (2000) who transplanted foetal striatal tissue pieces into five patients with Huntington’s disease and reported MRI and fluorodeoxyglucose PET evidence of graft survival, associated with improvement in cognition and mobility, in three of the five patients, whereas the two patients with no graft survival did not improve clinically. However, these effects appeared to wane by 10 years (Bachoud-Lévi *et al.*, 2000, 2006; Bachoud-Lévi, 2009) with some evidence of an ongoing low-grade immunological reaction (Krystkowiak *et al.*, 2007). A larger study, MIG-HD, undertaken by Bachoud-Lévi and colleagues between 2000 and 2012 was less successful than their pilot study in terms of survival of functioning grafts (Bachoud-Lévi *et al.*, 2020a). The processes in MIG-HD differed from the pilot trial in several important respects and raised issues relating to foetal tissue preparation, potential alloimmunization to graft material, surgical fidelity and trial design reviewed in Bachoud-Levi *et al.* (2020b), which have been key to the design of TRIal DEsigns for delivery of Novel Therapies in neurodegeneration (TRIDENT).

An alternative approach to preparing the donor foetal striatal promordia as tissue pieces is to prepare them as a cell suspension. The NEST-UK study (ISRCTN 36485475) used dissociated cells, demonstrating safety and feasibility in four participants who received staged bi-lateral striatal cell transplants and one who received simultaneous bilateral transplants with up to 12 million cells per side (Rosser *et al.*, 2002; Barker *et al.*, 2013). However, there was no clear, demonstrable functional improvement, perhaps reflecting the small participant numbers and the small graft deposits seen on imaging (Barker *et al.*, 2013), the latter suggesting that insufficient number of donor cells were transplanted (a consequence of the overriding safety concern at the time being potential tissue overgrowth). However, overgrowth was not observed, and the need for studies transplanting greater cell numbers has since become evident (in TRIDENT we plan to use the maximum used in NEST-UK as the minimum dose). Although the current clinical evidence provides some proof of principle that transplantation of foetal striatal progenitor cells may improve function in Huntington’s disease, the available data do not yet demonstrate it to be a robust and reliable therapeutic option, and thus further investigation is warranted. A future aim will be to replace foetal-derived cells with donor cells derived from a pluripotent stem cell source which will be more practically and ethically sustainable. However, at the time of writing, stem cell-derived products are not ready for clinical trial and hence a foetal-derived product provides the only cell source that has been documented for clinical application and which can be used to address clinical translation challenges and to gather further data on the safety and efficacy of CRT in Huntington’s disease.

The direct delivery of cells to the central nervous system is complex and presents several constraints which must be considered when designing CRT evaluations. First, the_current method by which cells are delivered to the target area uses an injection-based delivery system via a catheter/needle by which cells are deposited in beads along a preformed track as the delivery catheter is withdrawn (Torres *et al.*, 2015). However, much of the cell product is wasted, remaining within the large dead space volume of the catheter and much of the successfully delivered cells reflux back along the catheter track, leading to poor and uneven distribution of the transplanted cells. The development of improved devices and methods to combat these issues, along with their evaluation, are key components of trials evaluating CRT.

Second, the need to proceed with very small cohorts at this early experimental phase for safety reasons and the challenge of reducing study bias due to the ethical controversies around sham surgery are important considerations in the initial study design framework. Limited availability of sufficient foetal tissue of suitable quality dictates that transplants must occur sequentially and with significant time intervals. Furthermore, the effect of CRT on functional outcomes being a combination of the effectiveness of the cell-delivery device, the environmental integration of transplanted cells and the lag period between transplantation and any functional improvement being observed must also be considered.

Here, we present the protocol for the TRIDENT study (in recruitment at the time of writing) in which we plan to evaluate CRT in Huntington’s disease, with a view to developing innovative approaches that will minimize the impact of such constraints and maximize efficiency in future trials of CRT and similar therapies in Huntington’s disease and other related disorders.

## Methods

### Study design, setting and sample size

**Figure 1:**
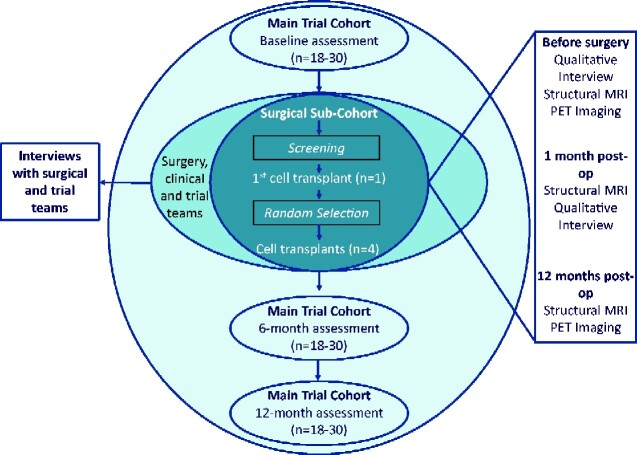
TRIDENT trial design.

TRIDENT is a phase I, single centre, Trial Within a Cohort (TWiC) (Relton *et al.*, 2010) designed to assess the safety and feasibility of increasing the number of human foetal cells transplanted (compared to the previous studies) intra-striatally into people with Huntington’s disease. It is recognized that evaluation of complex surgical interventions, such as CRT, may require an extended series of iterative pilot studies in preparation for randomized controlled trials (Craig *et al.*, 2008; Eldridge *et al.*, 2016). The design of this study has been guided by the output of the international workshop on trial design and ethics conducted as part of the REPAIR-HD consortium (Repair-HD Workshop Practicalities & Ethics of Trial Design). Key components of the design include longitudinal follow-up and selection of participants to receive the intervention using a trial within a cohort design, an in-depth deconstruction of the patient, carer and health professional experience at each stage of the process, leading to the development of a fidelity monitoring and health economic framework (Blencowe *et al.*, 2015). The TWiC design has been adopted to minimize bias introduced from an inability to blind the intervention and to use routinely collected [as part of an ongoing worldwide observational study (www.enroll-hd.org) assessment data as far as possible to reduce participant burden. We will recruit a minimum of 18 and maximum of 30 participants to form the TRIDENT observational cohort, from whom functional outcome data will be collected at specified time points (Fig. 1). A sub-set of the cohort will be approached to undergo pre-operative assessment to determine their suitability to receive the cell-transplant surgery (the surgical sub-cohort) and from this sub-cohort, up to five participants will receive the cell-transplant intervention. Participants selected to receive the cell transplant will undergo additional immunological and imaging assessments (outlined in further detail below) before and after the surgery. This study and surgery will be performed at University Hospital Wales, Cardiff, UK. Functional assessments will be completed at the South Wales Huntington’s disease clinic in Cardiff.

The sample size has been guided by the standard approach to phase I trials which restricts the number of participants being asked to take part in a highly novel, high-risk trial (McCulloch *et al.*, 2009), whereas allowing for a number of trial processes (such as randomization, surgical procedure and process evaluation) to be evaluated.

### Study objectives

The primary objective of TRIDENT is to evaluate the safety of transplantation surgery using a greater number of human foetal striatal cells than used previously for the treatment of people with Huntington’s disease.

Secondary objectives for the study include:

1. definition of a framework for assessing the fidelity of cell-transplantation procedures and surgical delivery devices;
2. exploration of effect estimates (through interrogation of functional and imaging outcome data to identify the variance of the measures across the whole cohort) to inform sample size calculations for future trials;
3. evaluation of the feasibility of a health economic evaluation for future trials;
4. exploration of attitudes and understanding, feasibility and acceptability of this process in Huntington’s disease patients and their family members/carers, trial deliverers and health professionals;
5. capture of the social experience of patients and family members/carers over the entire lifecycle of the cell-transplantation process, including the time period before, during and after the event;
6. identification of the support needs of patients undergoing neural transplantation and their family members/carers;
7. exploration of the expectations, attitudes and clinical equipoise of health professionals engaged in the activity of neural transplantation towards the transplantation process and trial processes (e.g. randomization).

### Recruitment and participant selection

Potential participants will be identified initially through the longitudinal study CAPIT-HD2 (Core Assessment Protocol for Intracranial Transplantation, developed in REPAIR-HD) (www.repair-hd.eu) and latterly the global platform study Enroll-HD (www.enroll-hd.org) via the regional Huntington’s disease clinic in Cardiff. Written information will be given to potential participants either at their routine clinic appointment or via letter prior to a clinic visit. All potential participants will be invited to discuss the study with the research team prior to giving informed consent.

Participants will be eligible for entry into the observational cohort if they meet all of the inclusion criteria and none of the exclusion criteria described below.

Inclusion criteria: (i) confirmed diagnosis of Huntington’s disease through genetic testing (CAG repeat length must be ≥39), (ii) ≥18 years of age, (iii) stage I or stage II disease (defined by a total functional capacity score, ≤12) and diagnosed as having motor symptom onset, (iv) participant is ambulatory and (v) participant must have capacity to provide informed consent.

Exclusion criteria: any ongoing major psychiatric disorder that would preclude the ability to take part in functional assessments or give informed consent.

Participants in the observational cohort will be reviewed by medically qualified members of the research team in line with the additional exclusion criteria for entry into the surgical sub-cohort outlined below. Those who are potentially eligible for inclusion in the surgical sub-cohort will be approached by the research team and will be invited for an in-depth discussion of the cell-transplant intervention with the chief investigators prior to informed consent being taken for pre-operative assessment.

Further exclusion criteria for entry into the surgical sub-cohort include: (i) the lack of a carer, significant other or family member to support attendance at regular assessments, (ii) ongoing use of anticoagulant medication, (iii) any significant medical condition that would compromise the safety of anaesthesia and/or surgery, (iv) deemed to be unsuitable for transplant surgery (e.g. inadequate striatal volume), (v) pregnancy and/or breastfeeding, (vi) previous immunizing event such as blood transfusion or previous transplant, (vii) contraindications to 3T MRI, (viii) contraindications to PET, (ix) any contraindication to immunosuppressive therapy, (x) any degree of chronic kidney disease, (xi) any positive blood test for HIV, Hepatitis B, Hepatitis C, active cytomegalovirus, active Toxoplasma Gondii, Human T-cell lymphotropic virus type 1, serology for active *Treponema pallidum* and (xii) females of child-bearing potential, or males with a partner of child-bearing potential, who will not commit to the prevention of pregnancy while enrolled in the study.

### Informed consent

Owing to the TWiC design with a sub-cohort nested within the main observational cohort, the trial will use a multi-stage consent model (Bibby *et al.*, 2018). Although there is evidence that people with HD can give valid consent to participation in an innovative and complex trial which is long-lasting (De Langavant *et al.*, 2015), the trial design meant that it was necessary to consent participants only to those procedures that they would be expected to undergo. All participants will be required to give written informed consent prior to enrolment in the observational cohort. Those participants asked to be included in the surgical sub-cohort will need to provide further written informed consent for the pre-operative screening assessments required for sub-cohort inclusion following further discussion of the study with the chief investigators. This conversation will be recorded, transcribed and a summary of the transcript will be sent back to the participant as an aide memoir for their decision making. Participants will be given a minimum of 2 weeks following this discussion to decide about their participation in the surgical sub-cohort. For the surgical sub-cohort, informed consent can only be obtained by the chief investigators. Finally, participants deemed suitable for surgery and who are selected to receive the cell transplant will be asked to provide written informed consent for the surgical procedure and assessments linked to having the surgery. This will be obtained by either of the chief investigators, but additional clinical consent will be obtained by the neurosurgical chief investigator performing the cell transplant prior to hospital admission in line with local clinical practice. At each stage, as part of the informed consent process, potential participants will be counselled about the inability to take part in other clinical trials where they have the possibility of receiving other novel potential disease-modifying therapies while participating in the TRIDENT trial. For participants who receive the CRT, this is likely to be a long-term consequence of participation in TRIDENT and this will be expressly noted prior to obtaining consent for surgery. For participants who remain in the observational cohort only, they will be free to take part in other clinical trials once their 12-month assessment has been completed.

Where family members or carers and trial delivery staff are requested to take part in qualitative interviews, individual written informed consent will be sought prior to interviews taking place.

### Study outcomes

The primary outcome measure for this trial will be safety at 4 weeks after surgery as defined by (i) the lack of incidence of significant additional, permanent neurological deficit, (ii) the lack of incidence of a clinically significant intra-cranial haemorrhage and (iii) the lack of incidence of clinically significant intra-cranial infection. All aspects of the primary outcome will be assessed and decided by the independent trial steering committee (TSC).

The secondary outcomes of this study will be; (i) feasibility and acceptability of clinical trial processes as determined by recruitment, retention and participant and carer experiences, (ii) fidelity of neurosurgery defined by the evaluation of successful delivery of cells and accurate neurological graft placement, (iii) long-term (12 months) safety of transplantation defined by growth profile of graft and the absence of development of clinically significant inflammatory and/or immune reaction, (iv) documentation of research, treatment and immunosuppression costs as well as feasibility of collection of patient-reported outcome measures to aid the development of a full-health economic evaluation in future trials and (v) development of fidelity markers through analysis of the surgical procedure and graft survival over 12 months.

### Observational cohort assessments

Participants in the observational cohort will undergo assessments at baseline, six (±2 weeks) and 12 months (±4 weeks) (Figure 1). At baseline and 12 months, this includes the SF-12 questionnaire of health-related quality of life (Ware *et al.*, 1996), the Client Service Receipt Inventory (CSRI) (Beecham and Knapp, 1992) and the CAPIT-HD2 (Supplementary Table 1) functional assessment battery (Table 1). CAPIT-HD2 was developed as part of the REPAIR-HD study (www.repair-hd.eu) and tests across motor, cognitive, psychiatric and functional domains using a number of widely used and validated measures as well as novel evaluations. At the 6-month time point, participants are assessed on only a truncated CAPIT-HD2 battery as summarized in Table 1. Owing to the nature of the intervention, assessments will not be blinded. However, to mitigate these limitations as far as possible, the Unified Huntington’s Disease Rating Scale (Huntington Study Group, 1996) will be recorded as video for rating by an independent assessor, and participants will wear a head covering to blind the assessor to their surgical status.

**Table 1:** CAPIT-HD2 assessment battery^a^

Name	Description
Motor domain	
Unified Huntington’s Disease Rating Scale (Total Motor Score)^b^^,^^c^	The Unified Huntington’s Disease Rating Scale is the gold standard measure for assessing motor severity in people with HD (Huntington Study Group, 1996)
Q-Motor^d^	
Speeded and metronome tapping^b^	Participants are required to tap their index finger on a force transducer according to cues. The duration and variability of finger taps are recorded (Reilmann and Schubert, 2017)
Dynamic cue and force matching	Participants are required to grip and lift a device fitted with a force transducer and hold it stable. Grip force, 3D position and orientation of the object are recorded (Medzech *et al.*, 2019)
Grasping and lifting	Participants are required to complete a series of tests where they generate force on a transducer with their index finger. They will be asked to; match force patterns for which they have previously received visual feedback, match a sinusoidal pattern, generate increasing and decreasing force patterns with and without visual feedback. Deviations from target forces and patterns are recorded
Q-Trail^d^	Participants are required to make a trail between specific numbers and/or letters using a stylus on a force transducer. Total distance travelled, total time used, precision of target identification (including total errors) and path precision are recorded
Q-Eye^c^^,^^d^	Participants are required to look at visual stimuli on a projected screen while their head is stabilized using a brow bar and chin rest. Eye movements (saccades, smooth pursuit and optokinetic nystagmus) in response to the stimuli are recorded
Cognitive domain	
Mattis Dementia Rating Scale^c^	The Mattis is a well-documented global measure of cognitive status, especially sensitive in sub-cortical affections (Mattis, 1976)
Hopkins Verbal Learning Test^c^	The HVLT is composed of 12 items, organized into three semantic categories and presented over three consecutive learning trials (Brandt, 1991)
Controlled Oral Word Association Tests (COWAT)	Participants are asked to name as many words (excluding proper nouns) beginning with a specific given letter (Loonstra *et al.*, 2001; Ardila *et al.*, 2006)
Category Fluency	Participants are asked to name as many things in one category as possible in a given time (usually 60 s) (Lezak, 1995)
Stroop test^b^^,^^c^	Participants are presented with a series of words pertaining to colours and are asked to read them out loud. Initially, the words are coloured to correspond to the word. Participants are asked to repeat the task with words written in contrasting colours, but they have to say the name of the colour the word is written in (Stroop, 1935)
Symbol Digit Modalities^b^^,^^c^	Participants are presented with a series of symbols and a code assigning a number (1–9) to each symbol. They have 90 s to write the corresponding number for the symbol for as many symbols as possible (Smith 2007)
Relationship and Life Events^d^	The relationship questionnaire is composed of 48 items. For each question, six possible responses are proposed: ‘absolutely true’, ‘true’, ‘mostly true’, ‘mostly false’, ‘false’ and ‘absolutely false’. The assessment of life events will be performed by using the Holmes & Rahe Scale (Holmes and Rahe, 1967). Patients are asked to tally 43 life events, which allows us to provide a score of events during the last year
Psychiatric domain	
Problem Behaviours Assessment^c^	This is a semi-structured clinical interview measuring the presence, severity and frequency of 11 key behavioural symptoms. Detailed severity scoring criteria are provided for each item (Craufurd *et al.*, 2001)
Apathy and Irritability Scales^c^	These are standardized questionnaires to assess apathy and irritability (Snaith *et al.*, 1978; Marin *et al.*, 1991)
Modified Frontal Systems Behavioural Scale	This is a brief, participant completed behaviour rating scale with demonstrated validity for the assessment of behaviour disturbances associated with damage to the frontal–subcortical brain circuits (Duff *et al.*, 2010)
Maze Task^c^^,^^d^	Participants are asked to make decisions when offered a choice between objects (decision making under limited choice) and when there is no list of options to select from (decision making under unlimited choice). Participants are told to make the decision as quickly as they can. The decision outcome is then recorded
Persistence Task^c^^,^^d^	This is intended to assess loss of motivation (an aspect of apathy). Participants are informed that they must race their icon against an opponent’s icon. They are also informed that their icon is fitted with a speed boost that the computer will activate at a random point in the race. Latency to quitting/completion is measured (McLauchlan *et al.*, 2019)
Functional domain	
Unified Huntington’s Disease Rating Scale Total Functional Capacity^c^	The Unified Huntington’s Disease Rating Scale TFC scale assesses how people with HD manage their work, finances, daily living, domestic chores and care arrangements (Huntington Study Group, 1996)
C3T^c^^,^^d^	The Clinch Token Transfer Test (C3T) is a dual-task assessment of bilateral, upper motor function that consists of three coin-transfer tasks which increase in difficulty (baseline simple, baseline complex and a dual task). The time taken to pick up and transfer the coins from the dominant to non-dominant hand and place into a purpose developed box is recorded. The addition of cognitive load increases the task complexity (Clinch *et al.*, 2018)

a All assessments are conducted at baseline and 12 months.

b Assessments included in the 6-month truncated assessment battery.

c Assessments required for the minimum data set.

d Novel assessment.

If the participant has performed an Enroll-HD assessment in the preceding 8 weeks to their TRIDENT assessment, data from common measures will be taken from Enroll-HD to prevent repeated exposure effects on cognitive assessments and minimize participant’s burden.

Observational cohort participants will be offered the opportunity to co-enrol in the HD-Clarity study (www.hdclarity.net), a global biobanking project collecting cerebrospinal fluid (CSF) from people with Huntington’s disease for biomarker analysis. Lumbar punctures to collect CSF would be performed at the baseline or 6-month assessments. For participants who then go on to have the CRT intervention, this CSF sample would be used for immunological marker analysis as the pre-operative baseline.

### Pre-operative screening assessments

Participants who are believed to be suitable to receive the cell-transplant therapy will be asked to consent to pre-operative screening to formally assess their suitability against the study inclusion/exclusion criteria. These assessments will be staged to minimize participant’s burden and risk. Blood tests to screen the health of participants will be performed. These will include standard pre-operative biochemistry and coagulation as well as a full virology and serology panel to determine the presence of infective agents (see exclusion criteria for detail). A blood sample will be retained for analysis of immunological markers in those participants who go on to have the CRT intervention. Serum pregnancy testing will be conducted in female participants. A 12-lead ECG will also be performed.

If the participant remains suitable for surgery after the blood tests and ECG, then they will undergo a 1.5T or 3T (if available) MRI under general anaesthetic. The scan serves two purposes: ensuring sufficient striatal volume for transplant and use for surgical planning. If the scan reveals insufficient striatal volume for transplant, then that participant will remain in the observational cohort.

### Participant selection for CRT

If participants have been deemed eligible for surgery through the pre-operative screening process, they will be considered to be in the surgical sub-cohort. From this sub-cohort, the clinical and surgical team will make a decision on the best first candidate in terms of clinical profile, social support and surgical safety to undergo the first CRT operation. Subsequent participants for CRT will be selected at random from the surgical sub-cohort to provide a direct assessment of willingness to be randomized to a CRT intervention. This will be done using a set of computer-generated random numbers to order the participants in the surgical sub-cohort. Where participants assessed for surgery either decline inclusion in the surgical sub-cohort or are found not to be suitable to proceed with the surgical intervention, they will remain as participants in the observational cohort.

### Cell-replacement therapy (study intervention)

Selected participants will be required to repeat baseline assessments if these were initially completed more than 6 weeks prior to surgery. A 3T MRI will be performed in the month prior to surgery for microstructure analysis and again at 1 month post-operatively. If the participant exhibits marked chorea, they may be given an anti-choreic medication prior to the scan to minimize movement artefacts. PET scanning using 18F-Fallypride will also be performed in the month prior to surgery to visualize striatal function. Urine pregnancy tests will be performed in female participants prior to PET scanning. Furthermore, a contrast CT angiogram will be performed in the month prior to surgery to visualize blood vessels for surgical planning.

#### Foetal cell preparation

Foetal cells for transplant are processed for use by the Cardiff Fetal Tissue Bank. This is a Human Tissue Authority accredited (Human Tissue Authority license no. 22639), REC approved (Ref 18/WA/0204), Good Manufacturing Practice facility licensed for the harvest and preparation of donated foetal tissue for cell transplant. Following informed consent potential maternal tissue donors are screened for presence of infectious agents prior to collection of tissue. foetal tissue and associated products are also tested for the presence of pathogens throughout the preparation process. foetal samples between 8 and 14 weeks gestation are collected and are staged developmentally using both intrauterine ultrasound measurements (pre-collection) and direct morphometric measuresments foetal parts (post-collection). Thus, although some variation in development age is inherent to this process, accurate data will be available for correlation with study outcomes. Due to the short time lines between collection and implantation, no characterization of the tissue is possible other than defining the gestational stage and ensuring sterility. Once collected, foetal tissue is transferred to the Cardiff Fetal Tissue Bank and whole ganglionic eminences are dissected for storage (maximum 7 days) in hibernation medium until the day of surgery. The process for preparing foetal tissue for transplant and the required sterility monitoring is detailed in Roberton *et al.* (2018). The resulting cell suspension is made to the required cell density and examined for viability (which must be ≥80% for transplant) before immediate transfer to the operating theatre for use within 8 h.

#### Cell-delivery device

To deliver the foetal cells to the striatum, a CE-marked device manufactured by Elekta (Stockholm, Sweden) with an in-house-manufactured inner cannula will be used. The Elekta outer cannula is a sterile stainless steel Backlund Injection device designed for use with the Leskell stereotactic frame and can be coupled with Luer-locked syringes for intra-cranial injection of substances. To our knowledge, the Elekta cannula has not, itself, been used for direct intracranial injection of cells previously although it was used as a guide cannula in the Reneuron^®^*Pisces* trials (Kalladka *et al.*, 2016). The inner cannula fits inside the Elekta device and can be coupled to the injection system. The inner cannula is single-patient use and will be pre-loaded with cell suspension prior to insertion into the outer Elekta cannula, and hence multiple inner cannulas can be used in each surgery depending on the number of donor cells available and injection tracts made.

#### Surgical procedure

The cell-transplant surgery will be conducted under general anaesthetic by an experienced consultant neurosurgeon (Co-Chief Investigator) highly familiar with sterotactic surgery and the apparatus used. Cannula trajectories and transplant co-ordinates will be calculated using the Neuroinspire^®^ navigation system with Neuromate^®^ (www.renishaw.com) robot guidance for manual advancement of the cannula to the target area. The inner cannula, pre-loaded with foetal cells will then be used to deliver them. Up to six tracts will be made per striatum (two in the caudate, two in the anterior putamen and two in the posterior putamen), based on striatal volume. Each tract will consist of up to five deposits of cells at 1–2 mm intervals as the needle is withdrawn. In the first surgery, a total of 12 million cells will be injected in a uni-lateral transplant.

The first participant will receive a unilateral transplant and, if this is deemed safe after a TSC review, we anticipate inviting this participant to undergo a similar transplant (same number of cells) in the other hemisphere with at least 6 weeks inbetween surgeries. The ideal time window between surgeries is not known. In their successful pilot study of five individuals, Bachoud-Lévi *et al.* performed staged surgical implantations a year apart. In the MIG-HD study, the gap between surgeries was only ∼4 weeks and few participants had good functional grafts, as judged by raclopride and fluorodeoxyglucose PET (Bachoud-Lévi *et al.*, 2020a), raising the possibility that this short gap increased the risk of alloimmunization (Bachoud-Lévi *et al.*, 2020b). However, the relationship between the existence of graft-associated antibodies and poor graft survival in MIG-HD was not clear and there were several other potential reasons for the poor graft survival (Bachoud-Lévi *et al.* 2020b). Given the theoretical possibility that alloimmunization is associated with an inter-surgical gap of just a few weeks, the precise gap is currently under review and may be guided by the presence of antibodies (in blood and CSF).

For subsequent participants, the CRT intervention may be delivered as a single-stage bi-lateral procedure, but will be based on clinical judgement of the surgical and clinical team with expert input from the TSC. It is anticipated that the second participant will receive an increase of 2.5 million cells per striatum and that this will continue with each subsequent surgery to a maximum of 22 million cells per striatum.

### Immunosuppression

Participants will undergo a period of immunosuppression that is expected to last at least 12 months (from the last surgery in the case of sequential uni-lateral transplants) to prevent graft rejection. The regime selected is a standard protocol for preventing donor tissue rejection after organ transplant and consists of: tacrolimus, 0.1 mg/kg for 12 months; mycophenolate mofetil, 500 mg twice daily for 12 months; prednisolone, 20 mg/day with staged reduction to be steroid free by 8-week post-surgery, cefotaxime on the day of surgery, co-trixamole, 480 mg/day for 6 months and nystatin for 14 days. Levels of tacrolimus will be monitored closely at the start of immunosuppression and throughout to achieve a consistent blood trough level of 4–7 μg/l. Dose modification of mycophenolate mofetil may be necessary if a participant develops neutropenia. Participants will be provided with a medication diary as a reminder to take the correct immunosuppression and compliance will be monitored at each follow-up visit (Supplementary Table 1). Restricted medications include any of those listed in the Interaction with other medicinal products section (section 4.5) of the summary of product characteristics of any of the immunosuppressive drugs listed above. Participants are permitted to continue with any medications prescribed for their disease as long as they are not in the list of restricted drugs. Female participants will undergo monthly urine pregnancy tests and, should pregnancy occur, we will follow local procedure for managing pregnancy in those on immunosuppression for solid organ transplant, where tarolimus is continued but dosage may need to be adjusted and mycophenolate is switched to azathioprine.

### Post-operative follow-up and assessments

Participants who have received the CRT intervention will be followed closely according to the schedule indicated in Supplementary Table 1. In addition to tacrolimus monitoring, blood will be analysed (biochemistry panel) to monitor health. Analysis of immunological markers [human leucocyte antigen and non-human leucocyte antigen antibodies, peripheral blood monocytes (phenotypic and functional analysis of T and B lymphocytes plus natural killer cells)] will be performed on blood and CSF samples taken at specified time points as indicated in the schedule of assessments (Supplementary Table 1). In the event that a participant needs to cease immunosuppression, additional CSF samples will be collected 1 month prior to and post-cessation of the immunosuppression. Urine pregnancy tests will be performed at least monthly in female participants on immunosuppression. One month after surgery transplant recipients will require a further 1.5T MRI scan to check graft placement. The 3T MRI scan and PET scan will be repeated at 12 months for microstructure comparison with the pre-operative scan and to assess functional integration of the graft, respectively. For all post-operative scans, if participants exhibit chorea likely to affect the image quality, then they will be given anti-choreic medication prior to the scan. Functional assessments will be performed at 6 and 12 months in line with those undertaken in the observational cohort. Adverse event and safety monitoring will be performed at each follow-up visit and all adverse events will be recorded. Any unexpected serious adverse events will be reported locally and to the appropriate ethics committee. Participant follow-up will continue after the end of the trial via the South Wales Huntington’s disease clinic.

### Monitoring

The primary objective of the trial, safety, will be reviewed and assessed by the TSC on a per-transplant basis. The TSC will consist of independent experts, covering the areas of neurosurgery, immunology, CRT and Huntington’s disease. The TSC will convene 4–6 weeks, following each surgery to review anonymized data (including pre- and post-operative MRI scans, operative and in-patient notes containing all blood results) from participants who have received CRT intervention to determine if the primary safety outcome has been met and if the trial should be halted for any reason. The TSC will be asked to pay specific attention to the following potential issues; the presence of a significant amount of material outside the target area of the striatum as visible on MRI, any abnormality on the post-operative scan that is linked to a functional deficit, any unexpected complication of surgery, to include any breaks, bends or blockages of the cell-delivery catheter or any other intra-operative or procedural concerns.

In addition to safety reviews on transplanted participants, the TSC will also be asked to review advances in the neural cell-transplantation field, with specific reference to cell-delivery systems. If it is felt that during the trial, a superior device for delivering cells to the target area of the striatum has become available, the interventional part of the trial will be paused. To make this happen, we would seek to re-start the trial using the superior device. Due to the small numbers involved, no additional data-monitoring committee will be convened.

### Participant’s withdrawal

Participants will have the right to withdraw fully or partially from the trial at any time. However, it will be made clear to participants going forward for surgery, once they have received the cell transplant, which they will need to remain in follow-up for safety reasons. However, participants would be permitted to withdrawal from further functional assessments.

### Process evaluation

All participants selected to undergo transplant surgery will be invited to take part in a series of brief semi-structured, in-depth interviews prior to and following their operation. Family members and/or carers of the participants may also be interviewed. The interviews will explore participants’ understanding of neural transplantation, perceptions and concerns and actual experiences of the cell-transplantation process. These interviews will also provide insight to key elements of trial design (feasibility and acceptability). A number of participants (anticipated sample size, 3–5) from the trial observational cohort will also be interviewed to gain their views and understanding on randomization, the consent process and participant materials, which can be contrasted with the views of those in the transplanted cohort.

Interviews will also be conducted with the health professionals involved in the recruitment and consent of participants, the surgical staff involved in the transplant process and with research staff involved in the completion of follow-ups and data collection. This will provide insight into trial design and feasibility, clinical equipoise and patient selection as well as views on CRT.

### Health economics

No formal health economic evaluation will be undertaken as part of this study but feasibility of collating health economic data in this participant population and for the CRT intervention will be assessed to inform future health economic evaluations in later efficacy trials. This will include the exploration of the feasibility, acceptability and sensitivity of collecting standard patient-reported outcome measures such as SF-12 compared to condition-specific questionnaires, collecting costing information during surgery to allow micro-costing of the intervention and using the CSRI to collect healthcare resource use information to estimate changes in healthcare cost in addition to implementation costs from both NHS and partial societal perspectives. Details of the health economic evaluation will be specified in the statistics and health economics analysis plan.

### Data management and confidentiality

All data will be collected and stored using a unique participant identification number to ensure confidentiality. All assessment data will be entered into the trial database with in-built range checks and data validation to ensure the accuracy of data collected. Where assessment data are to be obtained from Enroll-HD, this will be via an Enroll-HD-specific data request. Specific data management procedures are detailed in the trial data management plan.

### Statistical analysis

This study will involve descriptive analysis of clinical outcomes (Table 1) only. Continuous variables will be summarized as means and standard deviations, or medians and interquartile ranges if more appropriate, by sub-cohort (i.e. whether they did or did not receive CRT) and separately per time point (baseline, 6 and 12 months). Similarly, categorical variables will be summarized as frequencies and percentages, by sub-cohort and per time point. The group of participants who did not receive CRT will potentially be further divided into those who were initially selected and approached but did not receive the neural transplantation (e.g. refusal or ineligibility for surgery) and those who were never selected. In brief, 95% confidence intervals may be calculated for differences of group means or medians, but no formal statistical hypothesis testing will be performed.

Exploratory evaluations will be carried out to explore plausible trial designs for subsequent randomized trials evaluating the efficacy of neural transplantation in this population. Design considerations and parameters of interest will be detailed separately in the statistics and health economics analysis plan.

### Qualitative analysis

Transcribed interviews will be subject to framework analysis incorporating thematic and case analysis (Gale *et al.*, 2013) to allow for different data sources and diverse sampling. Minutes from trial management group meetings will also be included to identify themes pertinent to trial development and delivery. Agreement on concepts and coding between members of the research team will be sought and 20% of data will be coded by different team members to check coding scheme reliability. The complete description is given in the qualitative analysis plan.

### Ethics approval and consent to participate

Ethical approval for the study was given by Wales Research Ethics Committee 3 on 08/06/2018, Reference: 18/WA/0182. All participants must give full written, informed consent prior to inclusion in the study. This includes consent to participate for all qualitative interviews. All protocol amendments will be reported via ISRCTN.

### Data availability

Requests for access to trial data can be made to CTR@cardiff.ac.uk. The sharing of trial data will be pursuant to review of consent and contractual requirements.

## Discussion

The TRIDENT trial aims to assess the safety and feasibility of CRT [including the safety of transplanting a larger numbers of cells than used in our previous UK study (Rosser *et al.*, 2002)] for people with Huntington’s disease, while exploring a number of methodological issues to inform future trial designs. Key methodological, surgical and ethical challenges surrounding the investigation of CRT were identified through previous activities of the REPAIR-HD consortium (www.repair-hd.eu) and latterly through activities of the international networks Stem Cells for Huntington’s Disease and European Huntington’s Disease Network Advanced Therapies Working Group and Surgical Delivery Task Force (www.ehdn.org/advanced-therapies-wg). These challenges include: delivery device performance and surgical fidelity; methodology governing precise cell delivery; minimization of study bias; requirement for long-term follow-up (given the lag time between transplantation and measurable functional benefit being observed); limited availability of foetal tissue and the need to proceed cautiously with small clinical study cohorts for reasons of safety.

Here, we present a protocol for investigating CRT in Huntington’s disease whose design is centred around addressing some of the aforementioned constraints and challenges involved in CRT research. The TWiC design (Relton *et al.*, 2010), in which participants are randomly selected (following eligibility screening) to receive the surgical intervention from a pool of participants in whom longitudinal clinical data are collected, has been adopted for the benefits it offers in terms of efficiency (Van Der Velden *et al.*, 2017), pragmatism and potential reduction in disappointment bias (Relton *et al.*, 2010).

Given the ethical concerns inherent in sham surgery, achieving double blinding in CRT research is challenging. Although this is not a particular concern in this trial, as we are primarily concerned with surgical safety, we have taken steps to minimize bias as far as possible through masking of rater-administered assessments. Bias is being addressed further through ongoing work focussing on the development of quantitative clinical outcome measures using digitized objective assessments. There are, of course, limitations to this. For example, this study (and previous similar studies to date) does not control for the use of immunosuppressives that could theoretically have a neuroprotective effect (Matsumoto *et al.*, 2018; Jung and Yoon, 2020). Ultimately, these issues may impose the need for sham controls in the future, although it is also worth highlighting that instances of graft failure (i.e. a graft that does not survive) can be used to provide a comparison equivalent to sham surgery. Specifically, transplanted cells that fail to survive or integrate can provide no functional benefit and hence the participant would have been exposed to all the procedures associated with CRT without the benefit of a healthy surviving graft, in contrast to sham surgery which generally only reproduces some elements of the surgical process and stops short of brain penetration. For future trials where investigation of efficacy is the primary focus, it may be appropriate to perform sham surgeries and this would be judged on its merits at the time, and would require modification to the trial design.

Evaluation of CRT is likely to require a number of small, iterative pilot studies to assess safety and feasibility and explore effect estimates and study design aspects prior to moving forward with randomized controlled trials for assessing efficacy. Here, the use of the TWiC design promotes efficiency through the development of a cohort with periodic and longitudinal collection of functional outcome data with the possibility of cohort expansion across time. This, therefore, allows the possibility for testing more than one intervention, over time, within the cohort (Relton *et al.*, 2010). This is particularly advantageous in a rare disease such as Huntington’s disease where the number of potentially eligible participants for such interventions is limited. However, the degenerative nature of Huntington’s disease would require careful participant selection to ensure that those in the intervention cohort are sufficiently similar to those in the cohort acting as controls. Furthermore, the potential for longitudinal data collection (a defining feature of cohort studies) is attractive for studies investigating the safety and utility of novel procedures, and those using novel devices, as it allows for the ongoing collection of safety data which is a vital part of the regulatory pathway.

CRT may prove to be a beneficial therapeutic strategy in neurodegenerative disorders other than Huntington’s and Parkinson’s disease, particularly in those in which there is a relatively focal anatomical distribution of neuronal loss. As an autosomal dominant disorder with almost complete penetrance, Huntington’s disease can be diagnosed with confidence in life, making it a powerful paradigm for understanding and treating other neurodegenerative conditions. Thus, it is anticipated that identifying the principal requirements for successful CRT through this and future trials will eventually be applicable to similar research in other disorders of the central nervous system.

The complexities of addressing the challenges and constraints facing CRT research and how this can be translated into clinical practice in the future have highlighted the requirement for thorough and transparent reporting of studies of advanced therapies (to include CRT and other novel therapeutic strategies such as anti-sense oligonucleotides and gene therapies). The template for intervention description and replication (Hoffmann *et al.*, 2014) provides a framework for ensuring the comprehensive description of complex interventions such as CRT in the first instance to ensure transparency and allow replication. However, CRT comprises several critical interventional components, not least an intricate surgical procedure requiring significant experience and expertise, all of which need careful consideration as the field of advanced therapies research in Huntington’s disease and other neurodegenerative disorders develops and moves towards realistic therapies. To ensure consistency and comparison across studies with multiple therapeutic moieties, research teams and study designs, a framework for research progression should be followed. We suggest that an appropriate solution would be to develop an expansion to the *Idea, Development, Exploration, Assessment, Long-term follow-up* (IDEAL) framework (McCulloch *et al.*, 2009), originally developed to describe the stages of innovation in surgical process with recommendations on how to approach each stage. Since the inception of the IDEAL framework, it has been expanded to include guidance for innovation in physiotherapy (Beard *et al.*, 2018) and medical devices (Sedrakyan *et al.*, 2016) and could be adapted again to incorporate advanced therapies for neurodegenerative disorders. Having such a framework for development would enable consistency in the development of advanced therapies, thus enabling more straightforward comparison of novel interventions for the treatment of neurodegenerative disease.

A unique aspect of the TRIDENT trial is that, unlike many early-phase trials, it includes a comprehensive process evaluation as a core outcome. This is being undertaken at this early stage with the intention of documenting the intricacies of conducting CRT research from both the perspectives of participants involved and from those tasked with its delivery. We believe that understanding these processes as fully as possible is vital for developing robust methodology for future studies.

The inclusion of a process evaluation at this juncture also serves to highlight a fundamental aspect underpinning the design of TRIDENT, which has been to embed public and patient involvement (PPI) throughout. Many of the ethical and logistical challenges of pursuing research of this nature were voiced at the REPAIR-HD workshop on the practicalities and ethics of trial design (‘Repair-HD Workshop Practicalities & Ethics of Trial Design’) where invited PPI representatives were involved in the co-production of the workshop outputs. At this workshop, it was noted that participants and their families should be considered partners in the research and that researchers should be mindful that the study outcomes relate to what people with Huntington’s disease actually want. At all stages, we have engaged with our PPI partner with reference to study design and all logistical aspects of trial delivery, which has been facilitated by the presence of our PPI partner at regular trial management group meetings. In our experience, the inclusion of our PPI partner has proved an invaluable resource in keeping the participant voice at the forefront of what we are trying to achieve.

TRIDENT will test the safety of a potentially efficacious dose of primary foetal cells, giving us greater insight into the potential requirements for successful CRT. In the long term, it is unlikely that foetal cells will continue to be the primary source of tissue for CRT due to their limited availability, the ethical issues surrounding their use and the difficulties in standardizing processing according to Good Manufacturing Practice. Indeed, although the developmental stage of donor foetal tissue is limited to a specific gestational window (8–12 weeks, but in practice most tissue collections fall within an 8- to 10-week window), each participant will be effectively transplanted with a different cell product which may lead to variability in outcomes.

Replacing the donor cell source with striatal progenitor cells derived from human pluripotent stem cells is a plausible solution (Rosser and Svendsen, 2014). The generation of human pluripotent stem cells for use in CRT is an area of ongoing investigation in both Huntington’s disease (Li and Rosser, 2017) and Parkinson’s disease (Petit *et al.*, 2014) and their use has already been trialled, albeit in a small, uncontrolled safety trial, as a treatment for ischaemic stroke (Kalladka *et al.*, 2016) and the first stem-cell transplants trials for Parkinson’s disease have commenced. Importantly, the information and outcomes gathered in TRIDENT will be pivotal for establishing the principles and generating a robust methodological framework to undertake clinical assessment of the safety and efficacy of future stem-cell sources that have been selected through rigorous *in vitro* and *in vivo* laboratory testing.

## Trial status

This trial is currently open with recruitment to the observational cohort commencing on 10 August 2018 with plans to close recruitment to the observational cohort by March 2021. The trial is on version 5.0 (dated 8 May 2020) of the protocol. Results of the trial will be disseminated via peer-reviewed publication and ISRCTN. Additional communication of results will be made to trial participants.

## Supplemental material

Supplementary material is available at *Brain Communications* online.

## Supplementary Material

fcaa230_Supplementary_Data

## Abbreviations

CAPIT =: Core Assessment Protocol for Intracranial Transplantation
CSRI =: Client Service Receipt Inventory
CRT =: cell-replacement therapy
PPI =: public and patient involvement
REC =: research ethics committee
TRIDENT =: TRIal designs for DElivery of Novel Therapies for neurodegeneration
TSC =: trial steering committee
TWiC =: trial within a cohort
