# Supplementary material for: Protocol for an open label: phase I trial within a cohort of foetal cell transplants in people with Huntington’s disease
Source: Brain Commun. 2021 Jan 19;3(1):fcaa230. doi: 10.1093/braincomms/fcaa230 (PMC7850012; doi:10.1093/braincomms/fcaa230)
Supplement: fcaa230_Supplementary_Data [file fcaa230_supplementary_data.pdf]

Supplementary table 1. Schedule of assessments

Main Trial Cohort

	Enrollment		Month of Trial Participation												
	Up to -6 months		0	1	2	3	4	5	6	7	8	9	10	11	12
Discusion of trial	x														
Informed consent		x													
CAPIT-HD2 assessment battery			x												x
Truncated CAPIT-HD2 assesment battery									x						
SF-12 Quality of Life			x												x
Client Service Receipt Inventory			x												x
CSF collection (HD Clarity)(at either timepoint)			x						x						
Interview (optional)												x			

Surgical Sub-Cohort

	Enrollment and pre-op assessment										Month of post surgical follow up																																																
	-6 to -4			-3		-2		-1		0	1				2				3				4				5				6				7				8				9				10				11				12			
Number of visits per week	1			1		1	1	1	1			2	1	1		1			1				1				1				1								1								1								1			
Discussion of trial	x																																																									
Informed consent		x																																																								
Invitation to pre-operative assessment			x																																																							
Consent to pre-operative assessment				x																																																						
Blood Tests: biochemistry				x						x	x	x	x	x		x			x				x				x				x								x								x								x			
Viral serology				x						x																																																
Drug levels										x	x	x	x	x		x			x				x				x				x								x								x								x			
Pregnancy Test (Serum HCG)				x																																																						
Immunology Markers				x									x			x			x												x								x																x			
Urine pregnancy test							x			x				x					x				x				x				x								x								x								x			
ECG				x																																																						
1.5T MRI (1st under GA)						x								x																																											
CTA scan						x																																																				
Randon selection for surgery						x																																																				
Consent for surgery							x																																																			
3T MRI Scan								x																																																		
CAPIT-HD2 assessment battery								x																																														x			
Truncated CAPIT-HD2 assesment battery																															x																											
CSF Collection				x																											x																								x			
SF-12 Quality of Life								x																							x																								x			
CSRI								x																																															x			
PET Scan							x																																																x			
Interview									x					x																																												
CRT surgery										x																																																
Adverse event monitoring										x	x	x	x	x		x			x				x				x				x												x												x			
Immunosupressive drug compliance										x	x	x	x	x		x			x				x				x				x												x												x			

Denotes inpatient stay
